# Effectiveness of robotic rehabilitation interventions in children with cerebral palsy: protocol for a systematic review and meta-analysis of randomized controlled trials

**DOI:** 10.1186/s13643-026-03139-4

**Published:** 2026-03-03

**Authors:** Wei You, Kejimu Sunzi, Quanmin Deng, Lina Yin, Yang Gao, Yao Chen, Cheng Lei

**Affiliations:** 1 Emergency Department, Zigong Fourth People’s Hospital, Zigong, China; 2 Department of Pediatrics, Deyang People’s Hospital, Deyang, China; 3 Department of Whole Life Cycle Health Management Center, Deyang People’s Hospital, Deyang, China; 4 Nursing Department, Zigong Fourth People’s Hospital, Zigong, China; 5 Sichuan Clinical Research Center for Cancer, Sichuan Cancer Hospital & Institute, Sichuan Cancer Center, University of Electronic Science and Technology of China, Chengdu, China

**Keywords:** Robotic, Rehabilitation interventions, Children, Cerebral palsy

## Abstract

**Background:**

Cerebral palsy (CP) is the leading cause of childhood motor disability, often requiring intensive, long-term rehabilitation to improve motor function and independence in daily activities. Robot-assisted therapy has emerged as a promising intervention, as it can deliver high-intensity, task-specific, and motivating treatment. However, evidence for its superiority over conventional rehabilitation interventions (CRIs) is inconsistent, and a comprehensive quantitative synthesis is lacking. This protocol details a systematic review and meta-analysis to evaluate the effectiveness of robot-assisted therapy on motor outcomes in children with CP.

**Methods:**

This protocol has been developed in accordance with the Preferred Reporting Items for Systematic Reviews and Meta-Analyses Protocols (PRISMA-P) statement. We will conduct a comprehensive search of five electronic databases (PubMed, Embase, Web of Science, CINAHL, and the Cochrane Library) from their inception to June 2025. The review will include randomized controlled trials (RCTs) comparing robot-assisted therapy with CRIs in children with CP. Two independent reviewers will screen titles, abstracts, and full texts and extract relevant data. Primary outcomes will include measures of gross motor function, activities of daily living (ADL), gait, and balance. Secondary outcomes will include upper limb function and quality of life. Where appropriate, we will perform a meta-analysis using a random-effects model in Stata 14.0. We will investigate sources of heterogeneity using subgroup and sensitivity analyses. Publication bias will be assessed with funnel plots and Egger’s test. Furthermore, Trial Sequential Analysis will be employed to assess the robustness of the findings and control for risks of random error. The overall quality of evidence will be evaluated using the Grading of Recommendations, Assessment, Development and Evaluations (GRADE) framework.

**Systematic review registration:**

PROSPERO CRD420250652267.

**Supplementary Information:**

The online version contains supplementary material available at 10.1186/s13643-026-03139-4.

## Introduction

Cerebral palsy (CP) is the most common physical disability in children, affecting around 2 to 3 out of every 1000 live births worldwide [1, 2]. It is characterized by permanent movement and posture disorders caused by non-progressive brain lesions during development. This can result in delayed walking, abnormal gait, and limitations in independence [3]. CP is classified into different types based on clinical presentation, with spastic CP being the most common form, accounting for 85 to 91% of cases [4]. Gait, which is essential for children’s functional abilities and quality of life, is a key focus for intervention as it is linked to improved community participation. Children with CP also face challenges from musculoskeletal changes as they grow [5–8]. Managing pediatric gait disorders requires a variety of interventions, with evidence supporting different active and passive approaches [9–11]. However, traditional rehabilitation methods have difficulties such as high therapist workload and challenges in providing consistent, repetitive movements needed for motor learning [12].

Robot-assisted gait training (RAGT) has been increasingly used in pediatric rehabilitation, with devices like the Lokomat® being commonly utilized. RAGT provides repetitive, high-intensity training based on sensorimotor learning principles [13]. Studies have shown improvements in gait parameters, endurance, and functional task performance in children with CP [14, 15]. However, previous systematic reviews have conflicting results. Cortés-Pérez et al. found RAGT to be superior to routine therapy in improving gait speed and distance, while Conner et al. concluded that there was no significant advantage over standard care [16, 17].

These divergent conclusions suggest that the overall effectiveness of RAGT may not be uniform across all contexts. It is plausible that such discrepancies stem from significant clinical and methodological heterogeneity among the primary studies included in past reviews. For instance, the functional severity of participants, often stratified by the Gross Motor Function Classification System (GMFCS), may be a key factor, as interventions could have differential effects in ambulatory versus non-ambulatory children [18]. Furthermore, variations in intervention dosage (e.g., frequency and duration of training) [19] and the developmental stage of the children [20] could also contribute to the observed inconsistencies [21].

To address the previously discussed gaps in the literature, this systematic review and meta-analysis is designed to provide a more comprehensive, nuanced, and methodologically rigorous synthesis of the evidence. Our study aims to distinguish itself from and improve upon prior reviews in several key ways:

First, we will adopt a broader scope by including a wider range of robotic interventions and assessing a more comprehensive set of patient-centered outcomes, including the often-overlooked domains of hand function and quality of life. Second, to specifically resolve the inconsistencies in the existing literature, we will conduct pre-specified subgroup analyses to explore how treatment effects may differ based on crucial moderators like participants’ functional severity (GMFCS level), age group, and intervention intensity. This will help clarify for whom, and under what conditions, robotic therapy might be most effective. Finally, we will employ more advanced statistical methods, including Trial Sequential Analysis (TSA), to rigorously evaluate the sufficiency and conclusiveness of the cumulative evidence and to control for the risks of random errors.

Through this multi-faceted and enhanced approach, our study seeks to provide more precise and definitive evidence-based guidance for clinical practice, rehabilitation guideline development, and the design of future trials.

## Method

### Study design and objective

This study will be conducted and reported in accordance with the Preferred Reporting Items for Systematic Reviews and Meta-Analyses (PRISMA) 2020 statement [22]. The protocol was developed following the PRISMA-P 2015 guidelines [23–25] and has been registered in the PROSPERO database (CRD420250652267). This registration ensures transparency and prevents outcome reporting bias. The final manuscript will adhere to PRISMA 2020 standards for reporting results. Our objective is to perform a systematic review and meta-analysis of RCTs to quantitatively assess and synthesize evidence on the comparative efficacy of RRIs versus conventional rehabilitation interventions (CRIs) in children with CP.

### Eligibility criteria

#### Inclusion criteria

We will use the Population, Intervention, Comparison, Outcomes, and Study Design (PICOS) framework to define the eligibility criteria.

Participants (P): We will include studies involving children and adolescents (aged 0–18 years) with a confirmed clinical diagnosis of any type of CP. No restrictions will be placed on the Gross Motor Function Classification System (GMFCS) level. For studies that include both pediatric and adult participants, we will contact the authors to request disaggregated data for the pediatric cohort. If pediatric-specific data cannot be provided, the study will be excluded.

Intervention (I): The intervention group must have received any form of robot-assisted therapy aimed at improving motor function (e.g., upper limb, lower limb, or trunk control). 

Studies using hybrid interventions, where robot-assisted therapy is combined with conventional therapy, will be included. We plan a pre-specified subgroup analysis to explore the effects of these combined approaches compared to standalone robotic therapy.

Comparison (C): The comparison group must have received non-robotic interventions. This includes conventional rehabilitation (e.g., physical or occupational therapy), standard care, a placebo intervention, or no intervention.

Outcome (O): The selection and prioritization of our outcomes are guided by the World Health Organization’s International Classification of Functioning, Disability and Health (ICF) framework. This ensures our assessment is comprehensive, covering the direct effects of the intervention as well as its broader impact on a child’s life.

#### Primary outcomes

Primary outcomes will focus on the ICF domains of Body Function & Structure and Activity, as these are the direct targets of task-specific motor training provided by robotic therapy. All outcomes will be assessed post-intervention and at follow-up, where available.

1. Gross Motor Function: This includes measures of overall motor skills, walking, and balance. Outcomes will be assessed using validated tools, including but not limited to the Gross Motor Function Measure (GMFM-66/88), gait parameters (e.g., gait speed, stride length), and balance scales (e.g., Berg Balance Scale, Timed Up and Go test).
2. Upper Limb and Hand Function: This includes measures of arm and hand activity and dexterity. Assessments may include the Fugl-Meyer Assessment for Upper Extremity (FMA-UE), Box and Block Test, and classifications like the Manual Ability Classification System (MACS).
3. Activities of Daily Living (ADL): This includes measures of functional independence and self-care. Standardized assessments will include the Pediatric Evaluation of Disability Inventory (PEDI) or the Barthel Index.

#### Secondary outcomes

Secondary outcomes will focus on the ICF domain of Participation, representing the important downstream effects of improved motor ability on a child’s engagement in life situations.

Quality of Life and Participation: This will be assessed using validated patient- or parent-reported outcome measures, such as the Pediatric Quality of Life Inventory (PedsQL) or other similar instruments.

#### Data handling and prioritization for outcomes

To ensure consistency in data extraction and to minimize outcome reporting bias, we will establish a pre-specified hierarchy of measurement tools for each outcome domain. This hierarchy will be based on the instruments’ psychometric properties (e.g., validity, reliability) and their frequency of use in the field of pediatric cerebral palsy.

If a single study reports an outcome using multiple eligible instruments, we will extract data only from our pre-specified preferred instrument for the primary meta-analysis. For example:

1. For gross motor function, the Gross Motor Function Measure (GMFM) will be the preferred instrument.
2. For quality of life, we will prioritize data from the Pediatric Quality of Life Inventory (PedsQL).

If a study does not use our preferred instrument but uses another validated tool for the same domain, we will extract the data from that tool. The use of different instruments for the same outcome will be noted and explored as a potential source of heterogeneity in our analyses.

Study Design (S): Peer-reviewed published RCTs (including pilot RCTs) with a parallel-group or cross-over design. Cross-over trials will be included only if data from the first phase (before cross-over) are available to avoid carry-over effects.

Language: The search will be restricted to articles published in the English language due to limitations in translation resources.

### Exclusion criteria

Studies will be excluded if they involved: mixed populations without extractable pediatric CP-specific outcomes; robotic platforms employed exclusively for diagnostic assessment; publications with incomplete datasets critical for quantitative synthesis (including missing means/SD) or irretrievable full texts despite exhaustive retrieval efforts; trials where RRIs served only as minor adjunctive components (< 20% of therapeutic exposure); primary investigations exhibiting substantial methodological concerns as operationalized by > 80% high-risk domains in quality appraisal; and redundant publications.

### Ethical considerations

As this study is a protocol for a systematic review and meta-analysis based on previously published literature, it does not require formal ethical approval. However, we will assess and report on the ethical approval status and the use of informed consent in the primary studies included in our review, where this information is available.

### Search strategy

A comprehensive electronic search will be conducted across five international databases (PubMed, Embase, Web of Science, Cochrane Library, CINAHL) from inception through June 2025, utilizing a PICOS-derived framework wherein search syntax incorporates controlled vocabulary (MeSH/Emtree) and free-text keywords addressing: Participants (children with CP: “cerebral palsy,” “spastic diplegia,” “child,” “adolescent”), Interventions (RRIs: “robot-assisted therapy,” “Lokomat”), Comparisons (CRIs), Outcomes (motor function, activities of daily living, gait/balance, and clinical outcomes), and Study Designs (RCT). Table 1 presents an example of a search strategy via PubMed, and the full search strategies for all included databases are provided in Supplementary File 1. Supplementary searches will include backward/forward citation tracking of included studies and relevant systematic reviews, as well as direct author correspondence to obtain unpublished datasets or clarify ambiguities in reported outcomes.

**Table 1:** Search strategy example (PubMed)

Search step	Search query
#1	Cerebral Palsy [MeSH Terms]
#2	“cerebral pals*”[Title/Abstract] OR “Little disease”[Title/Abstract]
#3	#1 OR #2
#4	“Child”[MeSH Terms] OR “Infant”[MeSH Terms] OR “Adolescent”[MeSH Terms]
#5	child*[Title/Abstract] OR infant*[Title/Abstract] OR pediatric*[Title/Abstract] OR paediatric*[Title/Abstract] OR adolescen*[Title/Abstract] OR teen*[Title/Abstract] OR youth[Title/Abstract]
#6	#4 OR #5
#7	“Robotics”[MeSH Terms] OR “Rehabilitation”[MeSH Terms]
#8	(robot*[Title/Abstract] OR robotic*[Title/Abstract]) AND (rehab*[Title/Abstract] OR therap*[Title/Abstract] OR train*[Title/Abstract])
#9	#7 OR #8
#10	“randomized controlled trial”[Publication Type] OR “randomized”[Title/Abstract] OR “placebo”[Title/Abstract]
#11	#3 AND #6 AND #9 AND #10

### Selection of studies

**Fig. 1:**
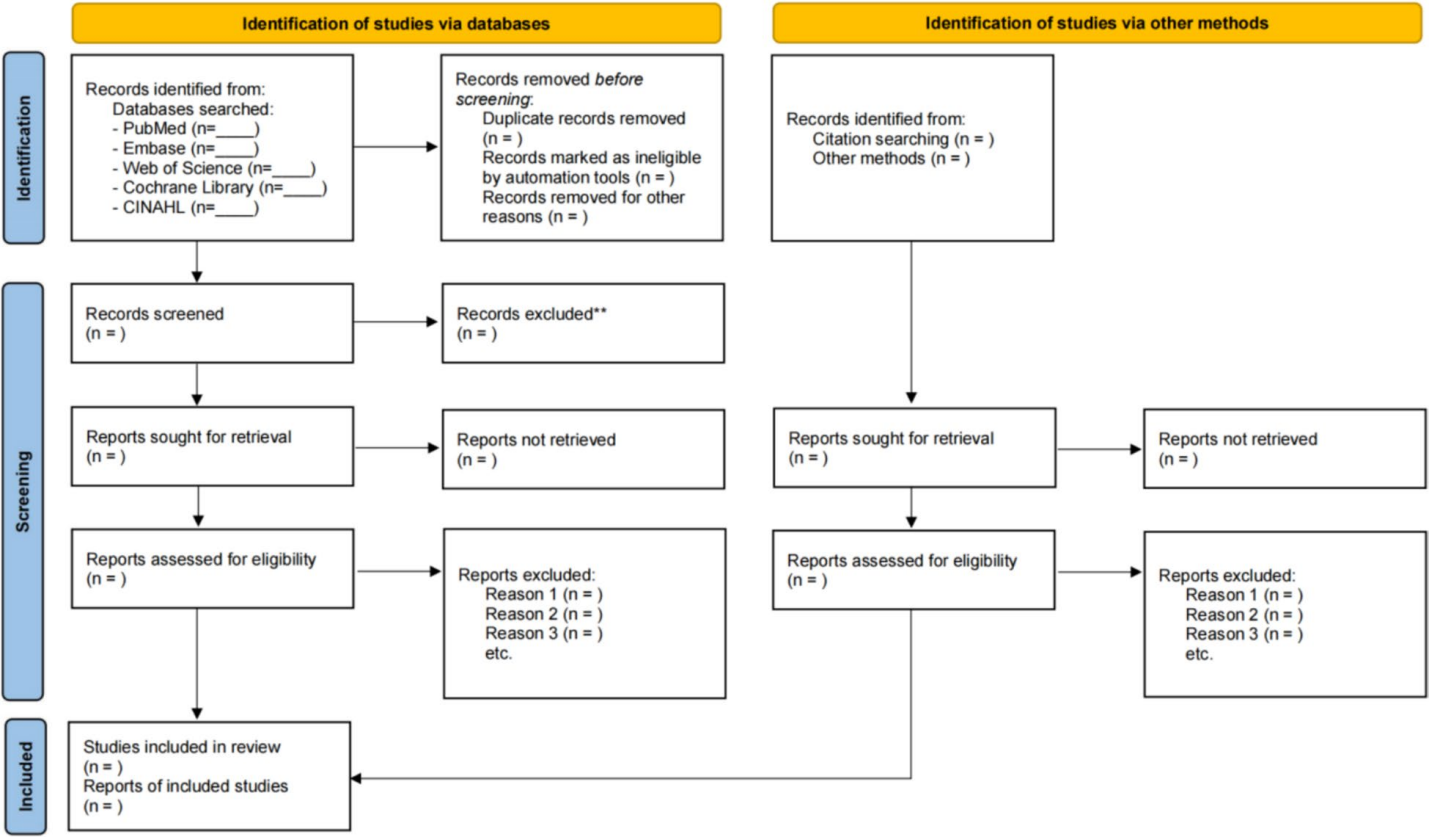
PRISMA flow chart depicting studies included in the systematic review and meta-analysis

Duplicate removal will be performed using EndNote X9 followed by a two-phase screening procedure: (1) blinded independent title/abstract assessment by two reviewers (KS, QD) employing standardized eligibility forms with pre-specified inclusion/exclusion criteria; (2) full-text appraisal of potentially eligible studies with comprehensive documentation of exclusion rationales, wherein inter-rater disagreements undergo arbitration by a third reviewer (CL) with final authority; inter-observer consistency will be quantified via Cohen’s Kappa coefficient (*κ* ≥ 0.8 target threshold), and the entire selection workflow—detailing identification, exclusion, and inclusion metrics—will be formally mapped in accordance with PRISMA standards using a flow diagram (Fig. 1).

### Data extraction

Two reviewers (KS, YG) will independently extract data from all included studies using a standardized data extraction form, which will be pre-piloted on a small sample of articles. Any discrepancies between the reviewers will be resolved through discussion or, if consensus cannot be reached, by consulting a third reviewer (CL).

The following data will be extracted from each study, organized into five key categories:

1. Study characteristics
  1. First author, year of publication, country of origin
  2. Study design details and sample size (per group)
  3. Funding sources and declared conflicts of interest
2. Participant characteristics
  1. Age (mean and range), sex distribution
  2. Cerebral palsy subtype and severity (e.g., GMFCS level)
3. Intervention and comparison details
  1. Intervention Group: Type of robotic device used, session frequency, duration per session, total duration of the intervention period, and details of any concomitant therapies
  2. Comparison Group: Detailed description of the conventional therapy or other control intervention, including its frequency, duration, and intensity
4. Outcome data
  1. Name of the outcome measure and time points of assessment (e.g., baseline, post-intervention, follow-up)
  2. For continuous outcomes: mean, standard deviation (SD), and number of participants (*n*) in each group
  3. For dichotomous outcomes: number of events and total number of participants in each group
5. Risk of bias information
  Information required to assess methodological quality using the Cochrane Risk of Bias 2 (RoB 2) tool.
  To ensure data extraction accuracy, a third reviewer (CL) will independently extract data from a random 10% sample of the included studies to verify inter-rater reliability.

### Risk-of-bias assessment

The methodological quality of each included RCT will be assessed by two independent reviewers (KS, WY) using the Cochrane Risk of Bias 2 (RoB 2) tool. Disagreements will be resolved through discussion or by a third reviewer (CL) to reach a consensus. The RoB 2 tool evaluates bias across five key domains: (1) the randomization process, (2) deviations from intended interventions, (3) missing outcome data, (4) measurement of the outcome, and (5) selection of the reported result. Each domain will be judged as having a “low risk,” “some concerns,” or “high risk” of bias, and the results will be presented in a summary figure.

Recognizing the unique challenges of rehabilitation technology trials, our assessment will also specifically consider the following potential sources of bias:

#### Performance and detection bias

Given the inherent difficulty of blinding participants and therapists to a large robotic device, we will critically evaluate the methods used to minimize bias, with a strong emphasis on whether outcome assessors were blinded.

#### Technology-related learning effects

We will examine whether studies implemented a sufficient familiarization or run-in period for both participants and therapists to mitigate the impact of initial inexperience with the technology influencing the results.

#### Intervention fidelity and device specificity

We will assess, where reported, how consistently the robotic intervention was delivered according to the study’s protocol. We will also extract details on the specific device used and its mode of application, as these factors can influence outcomes and the generalizability of the findings.

The updated Cochrane Risk of Bias Tool 2 (RoB 2) according to the instructions will be used for risk-of-bias assessment. The criteria for risk of bias will be assessed according to randomization process, deviations from the intended interventions, missing outcome data, measurement of the outcome, and selection of the reported result. Two researchers (KS and WY) independently evaluate using a blind method by hiding information such as the author and journal name. Disagreements will be resolved through consultation with a third researcher (CL). The consistency will be calculated by Cohen’s Kappa coefficient (*κ* ≥ 0.8 target threshold). The risk distribution of each bias domain will be reported as “high risk,” “low risk,” or “uncertainty” with summary figure and table in the results.

### Statistical analysis

All statistical analyses will be conducted using Stata 14.0. A *p*-value < 0.05 will be considered statistically significant unless otherwise specified.

#### Data synthesis and meta-analysis

We will perform meta-analyses using a random-effects model as the primary approach, given the anticipated clinical and methodological heterogeneity across studies. For continuous outcomes, effect sizes will be calculated as the mean difference (MD) with 95% confidence intervals (CIs) for outcomes measured on the same scale, and as the standardized mean difference (SMD) with 95% CIs for outcomes measured on different scales. For dichotomous outcomes, we will use the relative risk (RR) with 95% CIs.

#### Handling of multi-arm trials

Studies with multiple intervention or control groups will be handled according to the Cochrane Handbook for Systematic Reviews of Interventions. Our approach will depend on the nature of the trial arms:

If a study includes two or more similar intervention groups (e.g., different doses of robotic therapy) and a single control group, we will combine the intervention groups into a single group for the primary analysis, using established formulas to calculate a combined mean, standard deviation, and sample size.

If a study includes multiple, distinct intervention or control arms, we will only include the arms that are directly relevant to our review’s primary comparison (i.e., one robotic therapy arm vs. one conventional therapy arm). This ensures that each study contributes only one pairwise comparison to any single meta-analysis, thus avoiding the double counting of participants in a shared group.

#### Assessment of heterogeneity

Statistical heterogeneity will be evaluated using Cochran’s *Q*-test and quantified with the *I*^2^ statistic. The degree of heterogeneity will be interpreted in the context of the clinical and methodological diversity of the included studies.

### Subgroup and sensitivity analyses

To investigate potential sources of heterogeneity, we will conduct the following pre-specified subgroup analyses. These analyses will only be performed if each subgroup contains a minimum of three studies to ensure the comparisons are meaningful and to reduce the risk of spurious findings. The results of all subgroup analyses will be interpreted with caution.

1. Robotic Device Type: (e.g., gait-focused vs. upper-limb systems)
2. Intervention Intensity: (e.g., high-frequency vs. low-frequency)
3. Functional Severity of Participants: (e.g., GMFCS levels I–II vs. III–V)
4. Age Group: (e.g., < 6 years vs. 6–12 years vs. > 12 years)

We will assess the robustness of our findings through several sensitivity analyses. This will involve repeating the primary meta-analysis after excluding certain studies to test their influence on the overall result, specifically:

1. Studies judged to have a high risk of bias (as part of our standard sensitivity check).
2. Studies with a high rate of incomplete outcome data (e.g., attrition greater than 20%).
3. We will also apply the leave-one-out method, where one study is removed at a time to see its individual impact.

### Assessment of reporting biases

For outcomes reported in ten or more studies, we will visually inspect funnel plots for asymmetry and perform Egger’s statistical test to assess for publication bias. Furthermore, we will conduct Trial Sequential Analysis (TSA) to control for the risk of random errors and to determine whether the evidence is sufficient and conclusive.

### Certainty of evidence

Finally, the overall certainty of the evidence for each primary outcome will be independently assessed by two reviewers using the Grading of Recommendations, Assessment, Development, and Evaluations (GRADE) approach.

## Discussion

Robot-assisted rehabilitation has emerged as a promising technological intervention in pediatric rehabilitation. By offering high-intensity, task-specific, and engaging therapy, it aligns with established principles of effective motor learning and holds the potential to address the long-term needs of children with CP [13]. Despite this promise and a growing number of RCTs, the overall effectiveness of these interventions remains a subject of debate, with previous systematic reviews reporting conflicting findings [16, 17]. The present systematic review and meta-analysis was designed to provide a more comprehensive and methodologically rigorous synthesis of the current evidence to help clarify these inconsistencies.

A key strength of our review is its comprehensive scope. Unlike some prior reviews that focused predominantly on gait parameters [26–28], our study will synthesize evidence on a broader range of patient-centered outcomes, including the often-overlooked domains of hand function, activities of daily living, and quality of life. Furthermore, by conducting pre-specified subgroup analyses based on patient characteristics (e.g., GMFCS level, age) and intervention protocols, our review aims to move beyond a single overall effect estimate. This nuanced approach seeks to explore the sources of heterogeneity reported in earlier work and to identify for whom, and under what conditions, robotic therapy might be most beneficial [21, 29].

Despite its rigorous design, our review will have several potential limitations that warrant consideration. First, significant heterogeneity is anticipated across the included studies regarding the types of robotic devices, intervention protocols, and participant characteristics, which may limit the interpretability of pooled effect estimates. Second, while we are including only RCTs, the methodological quality of these trials may vary. Issues such as inadequate blinding or high attrition rates in some primary studies could introduce bias and affect the overall certainty of our evidence. Third, the potential for publication bias should be acknowledged. Smaller studies with positive findings are often more likely to be published than those with null or negative results, which could lead to an overestimation of the intervention’s true effect. Finally, the rapid evolution of robotic technology presents a unique challenge. Combining findings from older studies using first-generation devices with newer studies using more advanced systems may be complex, as the technology itself is a variable that is difficult to control for.

Should the evidence demonstrate a clear benefit, the findings of this review could have significant implications. For clinical decision-making, our subgroup analyses may provide more personalized guidance, helping therapists and families to identify which children (e.g., based on age or GMFCS level) might be the most suitable candidates for specific types of robotic therapy. From a broader health policy perspective, robotic interventions hold the potential to reduce the physical burden on therapists and improve access to high-intensity training, particularly in settings with limited human resources. However, these potential benefits must be weighed against significant practical challenges. The high capital cost of equipment, ongoing maintenance requirements, and the need for specialized training are substantial barriers to widespread implementation, especially in resource-limited environments. A balanced consideration of both the clinical effectiveness and the implementation challenges is therefore essential.

In conclusion, this systematic review aims to clarify existing controversies surrounding the use of robotic rehabilitation for children with CP. By providing a comprehensive synthesis of the evidence and a nuanced exploration of its limitations and implications, we intend for our findings to serve as a valuable resource for clinical practice, policy development, and the design of future research in this dynamic field.

## Supplementary Information

Additional file 1.

## Data Availability

The datasets used and/or analyzed during the current study are available from the corresponding author on reasonable request.
