# Supplementary material for: Effectiveness of robotic rehabilitation interventions in children with cerebral palsy: protocol for a systematic review and meta-analysis of randomized controlled trials
Source: Syst Rev. 2026 Mar 3;15:120. doi: 10.1186/s13643-026-03139-4 (PMC13063544; doi:10.1186/s13643-026-03139-4)
Supplement: Supplementary file 1 — Additional file 1. [file 13643_2026_3139_MOESM1_ESM.docx]

**1.Embase Search strategy**

#1 'cerebral palsy'/exp

#2 ('cerebral pals*':ti,ab OR 'little disease':ti,ab)

#3 (#1 OR #2)

#4 'child'/exp OR 'infant'/exp OR 'adolescent'/exp

#5 (child*:ti,ab OR infant*:ti,ab OR pediatric*:ti,ab OR paediatric*:ti,ab OR adolescen*:ti,ab OR teen*:ti,ab OR youth:ti,ab)

#6 (#4 OR #5)

#7 'robotics'/exp OR 'rehabilitation'/exp

#8 ((robot*:ti,ab OR robotic*:ti,ab) AND (rehab*:ti,ab OR therap*:ti,ab OR train*:ti,ab))

#9 (#7 OR #8)

#10 'randomized controlled trial':it OR 'randomized':ti,ab OR 'placebo':ti,ab

#11 (#3 AND #6 AND #9 AND #10)

**2.Web of Science (WoS) Search strategy**

(TS=("cerebral pals*" OR "Little disease")) AND (TS=(child* OR infant* OR pediatric* OR paediatric* OR adolescen* OR teen* OR youth)) AND (TS=(((robot* OR robotic*) AND (rehab* OR therap* OR train*)) OR robotics OR rehabilitation)) AND (TS=(randomized OR placebo) OR TI=(randomized OR placebo))

**3.Cochrane Library (Cochrane Central Register of Controlled Trials - CENTRAL) Search strategy**

#1 [mh "Cerebral Palsy"]

#2 ("cerebral pals*" OR "Little disease"):ti,ab,kw

#3 (#1 OR #2)

#4 ([mh Child] OR [mh Infant] OR [mh Adolescent])

#5 (child* OR infant* OR pediatric* OR paediatric* OR adolescen* OR teen* OR youth):ti,ab,kw

#6 (#4 OR #5)

#7 ([mh Robotics] OR [mh Rehabilitation])

#8 ((robot* OR robotic*) AND (rehab* OR therap* OR train*)):ti,ab,kw

#9 (#7 OR #8)

#10 (#3 AND #6 AND #9)

**4.CINAHL Search strategy**

#1 (MH "Cerebral Palsy+")

#2 (TI "cerebral pals*" OR AB "cerebral pals*" OR TI "Little disease" OR AB "Little disease")

#3 (S1 OR S2)

#4 (MH "Infant+") OR (MH "Child, Preschool+") OR (MH "Child+") OR (MH "Adolescent+")

#5 (TI (child* or infant* or pediatric* or paediatric* or adolescen* or teen* or youth) OR AB (child* or infant* or pediatric* or paediatric* or adolescen* or teen* or youth))

#6 (S4 OR S5)

#7 (MH "Robotics+") OR (MH "Rehabilitation+")

#8 (TI (robot* or robotic*) AND TI (rehab* or therap* or train*) OR AB (robot* or robotic*) AND AB (rehab* or therap* or train*))

#9 (S7 OR S8)

#10 (MH "Randomized Controlled Trials+") OR (TI (randomized or placebo) OR AB (randomized or placebo))

#11 (S3 AND S6 AND S9 AND S10)
